# A Cell-Free Biosensor for Assessment of Hyperhomocysteinemia

**DOI:** 10.1021/acssynbio.3c00103

**Published:** 2023-07-17

**Authors:** Fernanda Piorino, Shelbe Johnson, Mark P. Styczynski

**Affiliations:** School of Chemical & Biomolecular Engineering, Georgia Institute of Technology, 311 Ferst Drive NW, Atlanta, Georgia 30332-0100, United States

**Keywords:** hyperhomocysteinemia, homocysteine, folate deficiency, cell-free biosensor, diagnostics, monitoring

## Abstract

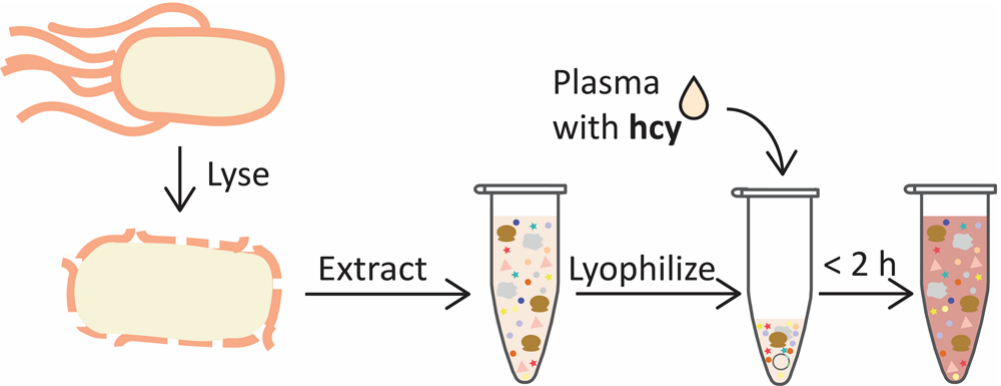

Hyperhomocysteinemia—a condition characterized by elevated levels of homocysteine in the blood—is associated with multiple health conditions including folate deficiency and birth defects, but there are no convenient, low-cost methods to measure homocysteine in plasma. A cell-free biosensor that harnesses the native homocysteine sensing machinery of *Escherichia coli* bacteria could satisfy the need for a detection platform with these characteristics. Here, we describe our efforts to engineer a cell-free biosensor for point-of-care, low-cost assessment of homocysteine status. This biosensor can detect physiologically relevant concentrations of homocysteine in plasma with a colorimetric output visible to the naked eye in under 1.5 h, making it a fast, convenient tool for point-of-use diagnosis and monitoring of hyperhomocysteinemia and related health conditions.

## Introduction

Hyperhomocysteinemia—abnormally elevated levels of the amino acid homocysteine in the blood—is linked to several diseases in the human body. For example, it is correlated with cardiovascular disease,^1,2^ cognitive impairment,^3^ cancer,^4^ homocystinuria,^5^ and B vitamin deficiencies.^6^ It is estimated to affect around 17% of the US population,^7^ but its prevalence can be as high as 38% in low-income countries.^8^ Hyperhomocysteinemia is most prevalent in low-resource areas where access to sophisticated diagnostic tools is limited, but homocysteine is typically measured via expensive methods that are often not available in those locations, including immunoassays,^9^ liquid chromatography–mass spectrometry, and high-performance liquid chromatography.^10^ A few research groups have developed alternative methods to assess homocysteine levels, but these methods either fail to detect clinically relevant levels^11^ or are not field-friendly.^12,13^ There is thus a need for an inexpensive, easy-to-use test to assess homocysteine status, which would be a powerful diagnostic and monitoring tool for a variety of health conditions.

A point-of-care homocysteine test would likely have its biggest and most global impact on the assessment of folate (vitamin B9) deficiency. Homocysteine is a functional biomarker for folate deficiency, with its plasma levels inversely related to folate status.^14^ Physiologically relevant concentrations of homocysteine typically fall between 5 and 100 μM, with healthy concentrations below 10–15 μM.^15,16^ Compared to the biomarkers more commonly used to assess folate deficiency—serum folate and red blood cell folate—plasma homocysteine samples are easier to process, more stable, and have more consistent reference concentration values.^17^

Folate deficiency was identified by the Biomarkers of Nutrition for Development program as one of six micronutrient deficiencies with the most significant global public health impact.^17^ Although fortification initiatives have helped to reduce the prevalence of folate deficiency to under 1%^7^ in the United States, folate intake is still inadequate in low-income, underdeveloped countries, and there is a lack of consistent population-based studies that specifically assess folate status to establish a consensus on the best indicators and concentration cutoffs.^18^ Additionally, public health interventions have failed to address the high prevalence of folate deficiency in women of reproductive age, who have higher folate requirements than the general population during pregnancy and while breastfeeding.^19^

In fact, folate status is a key metric for a healthy pregnancy as deficiency poses a risk to both the pregnant individual and the developing baby. Folate deficiency is associated with increased risk of congenital anomalies^20,21^ and anemia for the pregnant patient.^22^ Folate supplementation and monitoring of folate status are recommended from before conception to a few months postpartum, potentially creating financial and logistical burdens for patients; this further highlights the likely utility of a low-cost, easy-to-operate tool to assess folate status.

*Escherichia coli*-based cell-free biosensors have significant promise for satisfying the requirements of a broadly deployable homocysteine measurement tool. Cell-free biosensors detect a target analyte by combining an analyte-responsive genetic circuit and a bacterial cell extract containing the molecular machinery needed to express the components of that circuit and generate a reporter output.^23,24^ These sensors have been previously developed to detect a variety of analytes with outputs that can be easily interpreted with minimal equipment.^25−27^ They are inexpensive, easy to operate, and can be stored at room temperature once lyophilized, so they demonstrate great potential for point-of-use implementation.^28,29^ In addition, these sensors can reliably detect analytes in complex biofluids even after lyophilization.^25^

*E. coli* bacteria naturally sense homocysteine in their surroundings via a mechanism that involves the transcription factor MetR. Homocysteine binds to MetR to activate transcription from the P_GlyA_^30^ and P_MetE_^31,32^ promoters. This MetR-homocysteine-induced activation could potentially be used as a basis for a homocysteine sensing platform.

Here, we describe the development of a cell-free biosensor that harnesses the native homocysteine sensing machinery of *E. coli* to detect homocysteine in plasma samples. We show that our sensor can detect clinically relevant concentrations of homocysteine with a colorimetric output visible to the naked eye in under 1.5 h.

## Results

**Figure 1:**
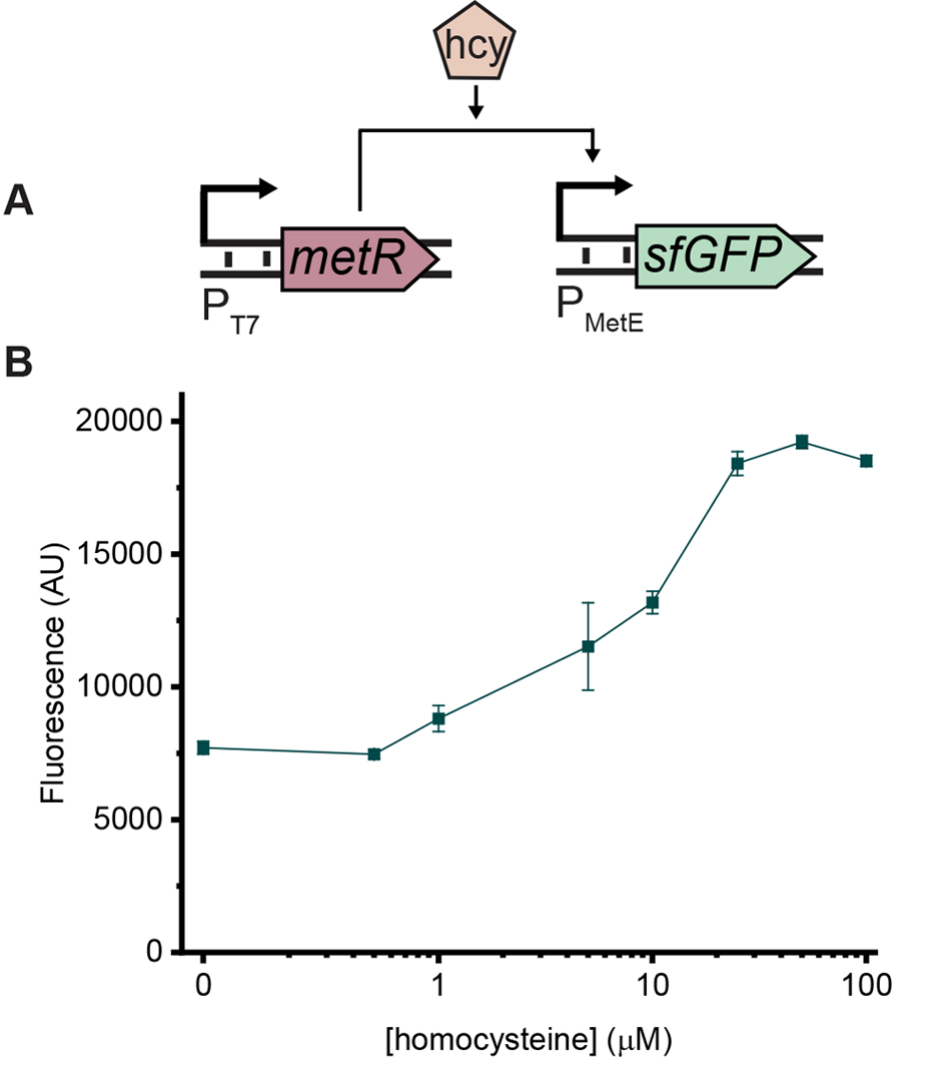
Proof of principle for a cell-free homocysteine sensor. (A) Schematic of the constructs used in the sensing circuit. In pMetR (left), a T7 promoter drives the expression of MetR. In pMetEGFP (right), P_MetE_ drives superfolder GFP (sfGFP) expression. Homocysteine (hcy) binds to MetR to activate expression from P_MetE_. (B) Dose response to homocysteine. One μM homocysteine induces GFP expression above background levels. Concentrations over 25 μM yield a saturated response. Reactions contain 10 nM pMetEGFP and 5 nM pMetR. Data were collected after 4 h of incubation at 37 °C. Background fluorescence was subtracted from all samples; error bars indicate the standard deviation of three technical replicates.

We engineered a homocysteine-responsive circuit distributed across two plasmids (Figure 1A). One plasmid, pMetR, constitutively expresses the transcriptional activator MetR from the P_T7_ promoter. The second plasmid, pMetEGFP, expresses superfolder GFP (sfGFP) from P_MetE_, an endogenous promoter regulated by the MetR-homocysteine complex. P_GlyA_ is another promoter regulated by this complex that was tested for use in this circuit, but it yielded an unacceptably high baseline expression of sfGFP and a higher limit of detection (Figure S1). We used sfGFP as the sensor’s reporter protein only for preliminary characterization of the sensing circuit.

When tested with clinically relevant concentrations of homocysteine (Figure 1B), the sensor exhibited a detection limit of 1 μM homocysteine, which is below the lower limit of the reference range. Fluorescence plateaued at 25 μM homocysteine, which is above the healthy reference range, resulting in a 2.58-fold induction relative to the reaction with no homocysteine added (Figure S2). This increase in fluorescence at high homocysteine levels occurred only in the presence of pMetR, demonstrating MetR-mediated activation of P_MetE_ (Figure S3).

We also assessed the specificity of the sensor for homocysteine compared with other molecules with similar structures that might be present in human plasma (Figure S4). In defined chemical mixtures, only one of the three molecules tested resulted in an increase in sensor output, though only at a concentration far above (almost 1000 times) its physiologically relevant levels.^33^ These results suggest that the sensing platform is reasonably specific to homocysteine and may be robust even in a complex biofluid matrix in which similar molecules are present.

**Figure 2:**
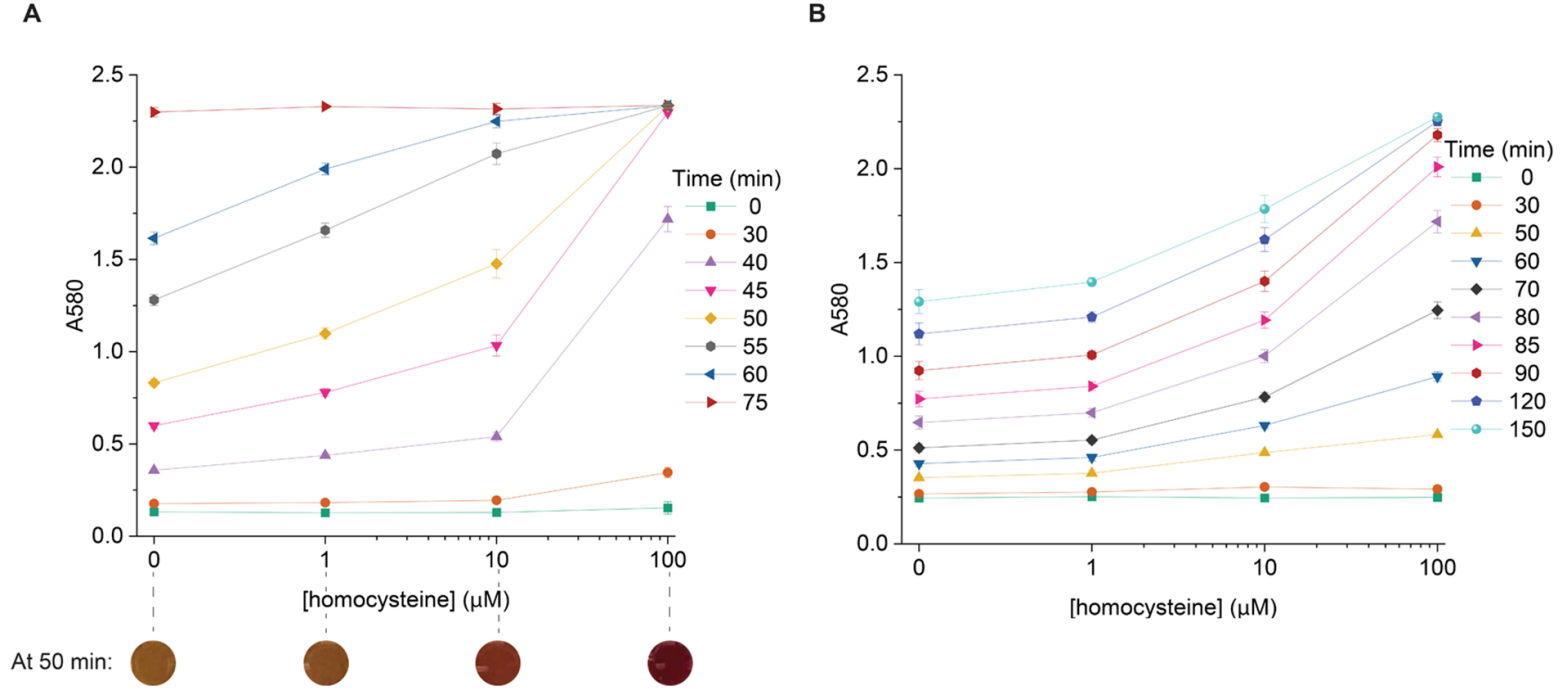
Toward a field-friendly cell-free homocysteine biosensor. (A) Colorimetric response in aqueous medium. Optimal response, with maximal differences in absorbance between successive measured concentrations, is observed around 50–55 min and is depicted in the images below the *x* axis. All reactions contain 0.83 nM pMetR and 1.67 nM reporter plasmid expressing LacZ. (B) Freeze-dried sensor response to homocysteine in 20% human plasma. Optimal response is slower (around 80–90 min). All reactions contain 3.33 nM pMetR and 6.67 nM reporter plasmid. Error bars indicate the standard deviation of three technical replicates. Homocysteine concentrations are the concentrations added to the plasma sample before dilution and measurement.

To make the biosensor more field-friendly, we replaced sfGFP with LacZ, a colorimetric reporter previously used in other cell-free sensors.^26^ The LacZ (β-galactosidase) enzyme converts a yellow molecule (chlorophenol red-β-d-galactopyranoside) to a purple molecule, causing an increase in absorbance at 580 nm and generating a semiquantitative readout with multiple intermediate colors discernible to the naked eye. We reduced plasmid concentrations to amplify the differences in absorbance and thus color between homocysteine concentrations (Figure S5). Differences in absorbance were maximal around 50–55 min (Figure 2A, with pictures of the reactions displayed below the graph); while distinguishing intermediate levels of homocysteine with the naked eye may be challenging, a more binary distinction between healthy (<10 μM, yellow-orange color output) and high levels of homocysteine (red-purple color output) should be easier to identify and would have diagnostic utility. If quantitative assessment is desired but difficult with the naked eye, use of a smartphone app to measure RGB values could be used to accomplish that task.^34^

With a colorimetric reporter established, we demonstrated sensor compatibility with two additional criteria for field deployment of the sensor: (1) measurement in plasma and (2) use of a lyophilized reaction. Figure 2B shows the detection profile at different reaction times in lyophilized reactions that were rehydrated with pooled human plasma samples containing different levels of homocysteine. Compared to detection in aqueous medium (Figure 2A), the freeze-dried sensor response in 20% plasma was slower even after adjusting plasmid concentrations, with maximal differences in absorbance observed around 90 min. In addition, it had slightly higher baseline colorimetric absorbance at 0 min (likely due to the absorbance of the plasma). Moreover, reactions with lower concentrations of homocysteine did not reach a saturating absorbance value of around 2.2 even after 150 min, even though in defined aqueous medium all concentrations reached saturating absorbance within 75 min. This potentially extends the readout window of the sensor, as colorimetric differences between reactions with low and high homocysteine levels may remain visible for longer.

To further validate that detection is robust in clinical samples, we also tested the sensor’s response to homocysteine in single-donor clinical serum samples (Figure S6). Overall, sensor response was similar to that observed in pooled human plasma (Figure 2A), but it was slightly faster, with maximal differences in absorbance occurring around 40 min. At that time point, each donor sample exhibited a characteristic response curve, likely due to inherent differences in homocysteine levels as well as sample matrix effects that could be addressed via a previously reported sample-specific calibration scheme.^26^

## Discussion

Here, we have demonstrated the feasibility of implementing a MetR-based, cell-free biosensor for the fast, inexpensive detection of physiologically relevant levels of homocysteine in plasma. Our platform generates color outputs, with an optimal detection time of around 90 min. In addition, the colorimetric readout did not require equipment to be interpreted: it was visible to the naked eye, certainly for qualitative assessment and perhaps for semiquantitative assessment. Use of a smartphone app to measure RGB values of the reaction’s color output could enable a fully quantitative interpretation without specialized equipment.

Ongoing work is necessary to demonstrate a fully fieldable sensor. For example, additional optimization can improve the time needed for sensor response and its linear dynamic range, and a final field-friendly form factor for the device (e.g., paper or a plastic housing) should be established. Nonetheless, the cell-free homocysteine biosensor reported here could be a powerful tool for the point-of-use diagnosis of hyperhomocysteinemia and related diseases. It can be operated by untrained users with minimal equipment and requires only droplet-sized volumes of blood, making it potentially usable even by patients in their own homes. This would further facilitate healthcare monitoring, such as of folate status during pregnancy, eliminating the need for multiple trips to a medical clinic, and broadening access to healthcare in areas where monitoring of homocysteine is most needed.

## Methods

### Materials

T5 exonuclease, Taq ligase, Phusion polymerase, Q5 polymerase, and restriction endonucleases were purchased from New England Biolabs (Ipswich, MA, USA). E.Z.N.A. Plasmid Mini and Midi Kits were purchased from Omega Biotek (Norcross, GA, USA), and QIAquick PCR Purification Kits were purchased from QIAGEN (Valencia, CA, USA).

### Strains and Plasmids

*Escherichia coli* K12 DH10B (New England Biolabs, Ipswich, MA) was used for plasmid assembly. *E. coli* BL21 Star (DE3) Δ*lacZ* was used to prepare the extract for cell-free expression. The plasmid pJL1, with a ColE1 origin and kanamycin resistance cassette, was used as the backbone vector for all plasmids.

### Cloning and Construct Assembly

All constructs were assembled with Gibson assembly.^35^ LB medium composed of 10 g/L NaCl, 5 g/L yeast extract, and 10 g/L tryptone was used for all cell growth during the cloning steps. Kanamycin (30 μg/mL) was used as appropriate for selection. The coding sequences for the *metR* gene and for the homocysteine-responsive promoter were isolated from DH10B genomic DNA, and the coding sequence for *sfGFP* was amplified from the plasmid pJL1. All plasmid sequences are described in the Supporting Information.

### Preparation of Cellular Lysate

Cellular lysate for all experiments was prepared as previously described.^36^ BL21 Star (DE3) Δ*lacZ* cells were grown in 2× YTP medium at 37 °C and 180 rpm to an OD of 1.7, which corresponded with the midexponential growth phase. Cells were then centrifuged at 2700 rcf and washed three times with S30A buffer. S30A buffer contains 50 mM tris, 14 mM magnesium glutamate, 60 mM potassium glutamate, and 2 mM dithiothreitol, and is pH-corrected to 7.7 with acetic acid. After the final centrifugation, the wet cell mass was determined, and cells were resuspended in 1 mL of S30A buffer per 1 g of wet cell mass. The cellular resuspension was divided into 1 mL aliquots. Cells were lysed using a Q125 Sonicator (Qsonica, Newton, CT) at a frequency of 20 kHz, and at 50% of amplitude. Cells were sonicated on ice with three cycles of 10 s on, 10 s off, delivering approximately 300 J, at which point the cells appeared visibly lysed. An additional 4 mM of dithiothreitol was added to each tube, and the sonicated mixture was then centrifuged at 12,000 rcf and 4 °C for 10 min. The supernatant was removed, divided into 1 mL aliquots, and incubated at 37 °C and 220 rpm for 80 min. After this runoff reaction, the cellular lysate was centrifuged at 12,000 rcf and 4 °C for 10 min. The supernatant was removed and loaded into a 10 kDa MWCO dialysis cassette (Thermo Fisher). Lysate was dialyzed in 1 L of S30B buffer (14 mM magnesium glutamate, 60 mM potassium glutamate, 1 mM dithiothreitol, pH-corrected to 8.2 with Tris) at 4 °C for 3 h. Dialyzed lysate was removed and centrifuged at 12,000 rcf and 4 °C for 10 min. The supernatant was removed, aliquoted, and stored at −80 °C for future use.

### Cell-Free Reactions

Cell-free reactions for all experiments were run as previously described.^37^ Each cell-free reaction contained 0.85 mM each of GTP, UTP, and CTP, in addition to 1.2 mM ATP, 34 μg/mL folinic acid, 170 μg/mL *E. coli* tRNA mixture, 130 mM potassium glutamate, 10 mM ammonium glutamate, 12 mM magnesium glutamate, 2 mM each of the 20 standard amino acids, 0.33 mM nicotine adenine dinucleotide (NAD), 0.27 mM coenzyme-A (CoA), 1.5 mM spermidine, 1 mM putrescine, 4 mM sodium oxalate, 33 mM phosphoenol pyruvate (PEP), 27% cell extract, and the specified plasmid concentrations.

Cell-free reactions were run in 10 μL volumes in 384-well small volume plates (Greiner Bio-One), and a clear adhesive film was used to cover the plate and prevent evaporation. Plates were incubated for 4 h at 37 °C, and fluorescence was measured with a plate reader (Synergy4, BioTek). Excitation and emission for sfGFP were recorded at 485 and 510 nm, respectively. For each set of reactions, three technical replicates were run using lysate from the same batch. Variability in reaction outputs across different batches of lysate was assessed, with results reported in Figure S7.

In reactions producing β-galactosidase, CPRG was added to a final concentration of 0.6 mg/mL. Reactions in plasma contained 20% pooled human plasma (Apheresis derived, Innovative Research Inc.) and ribonuclease (RNase) inhibitor (New England Biolabs) at 0.6 U/μL. Reactions in serum contained 20% previously collected and processed samples^26^ or pooled human serum purchased from MP Biomedicals.

### Lyophilization

Cell-free reactions were prepared as described in PCR tubes. Tubes were flash-frozen in liquid nitrogen and kept at −80 °C. Frozen tubes were transferred to a prechilled Labconco Fast-freeze flask. Flasks were connected to a Labconco FreeZone benchtop freeze-drier and lyophilized at −50 °C and 0.05 mbar for 3 h. Tubes were then removed and immediately recapped with a new lid. Tubes were stored in a sealed bag at room temperature until testing.

Lyophilized reactions were rehydrated in 33 μL of nuclease-free water containing 20% plasma, in which the plasma was supplemented with the specified concentration of homocysteine. The reactions were aliquoted and immediately transferred to a plate reader at 37 °C.

### Data Processing and Statistical Analysis

For cell-free experiments, all reported fluorescence values are background subtracted using the fluorescence of a reaction containing no plasmid.

The limit of detection was determined by comparing the fluorescence values of reactions with a specified homocysteine concentration with those of reactions with no added homocysteine using a two-tailed Student’s *t* test, assuming equal variance. The lowest concentration of homocysteine that yielded a *p*-value below 0.05 for that concentration and all higher concentrations was considered the limit of detection.

## Supporting Information Available

Supplementary Figures S1–S7: Dose–response of a sensor harnessing P_GlyA_ regulation, fold change in fluorescence intensity upon addition of homocysteine, characterization of the sensor system, specificity of the homocysteine biosensor, tuning of plasmid levels to improve colorimetric sensor response, colorimetric sensor response to single-donor serum samples, batch variability in cell-free homocysteine sensing; Supplementary Tables S1–S2: Annotated sequences of all plasmids, primer sequences (PDF)
