# Supplementary material for: A Cell-Free Biosensor for Assessment of Hyperhomocysteinemia
Source: ACS Synth Biol. 2023 Jul 17;12(8):2487–92. doi: 10.1021/acssynbio.3c00103 (PMC10443029; doi:10.1021/acssynbio.3c00103)
Supplement: Supplementary file 1 — sb3c00103_si_001.pdf [file sb3c00103_si_001.pdf]

Supporting Information for
A Cell-Free Biosensor for Assessment of Hyperhomocysteinemia

Fernanda Piorino, Shelbe Johnson, and Mark P. Styczynski*

School of Chemical & Biomolecular Engineering, Georgia Institute of Technology, 311 Ferst Drive NW, Atlanta, Georgia 30332, USA
*(Email: mark.styczynski@chbe.gatech.edu)

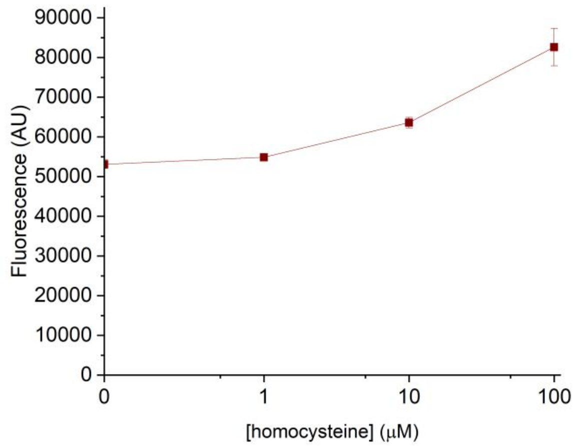

Figure S1. Sensor response with sfGFP expression driven by P_{GlyA} instead of P_{MetE}. The increase in fluorescence signal at 1 μM homocysteine is not statistically significant. In addition, the baseline fluorescence signal at 0 μM homocysteine is high, yielding a maximal fold increase in fluorescence of 1.55. With P_{MetE}, that value was 2.58 based on the data in Figure 1B. Background fluorescence of cell-free reactions with no DNA template was subtracted from all samples, data were collected after 4 hours of incubation at 37°C, and error bars indicate the standard deviation of three technical replicates.

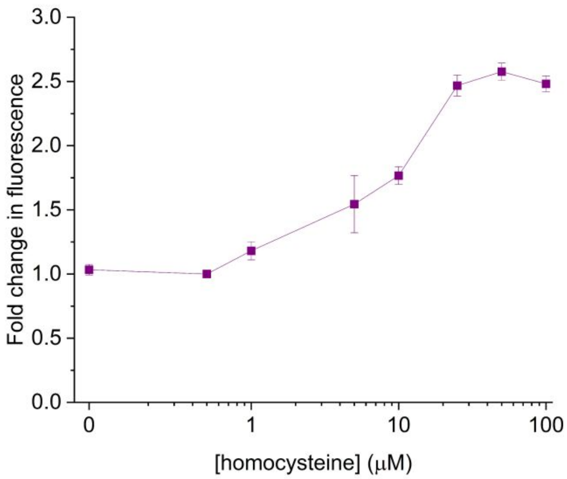

Figure S2. Fold change in fluorescence intensity upon addition of homocysteine relative to the lowest fluorescence output, with data reproduced from Figure 1B. Background fluorescence of cell-free reactions with no DNA template was subtracted from all samples, data were collected after 4 hours of incubation at 37°C, and error bars indicate the standard deviation of three technical replicates.

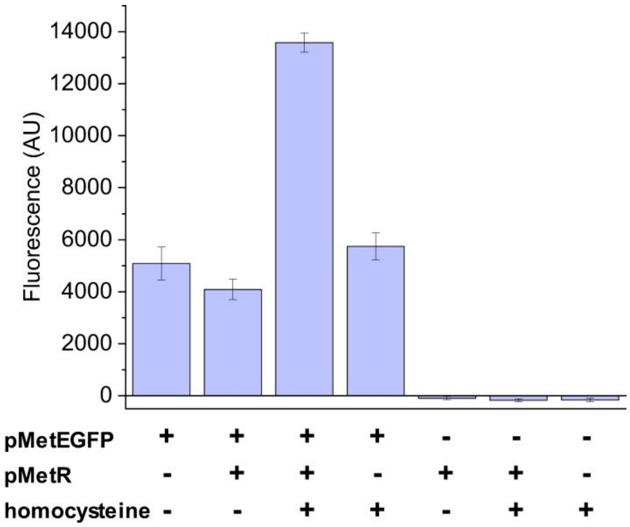

Figure S3. Characterization of the sensor system. The effect of individual and combinatorial addition of pMetEGFP (10 nM), pMetR (5 nM), and homocysteine (100 μM) was determined via sfGFP measurements. When pMetR is present, the system shows a strong response to homocysteine. Background fluorescence of cell-free reactions with no DNA template was subtracted from all samples, data were collected after 4 h of incubation at 37 °C, and error bars indicate the standard deviation of three technical replicates.

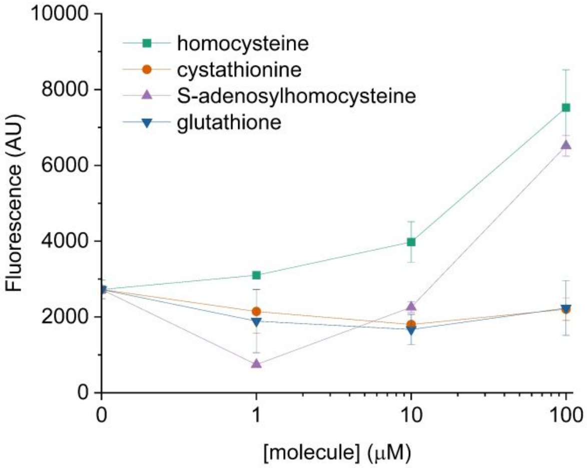

Figure S4. Specificity of the homocysteine biosensor. Out of three molecules similar in structure to homocysteine that may also be present in human plasma, the sensor has no response to two of them and only responds to S-adenosylhomocysteine at levels that are three orders of magnitude greater than the physiologically relevant concentrations that would be observed in blood samples.

Each reaction contains 10 nM of pMetEGFP and 5 nM of pMetR. Background fluorescence was subtracted from all samples, data were collected after 4 h of incubation at 37 °C, and error bars indicate the standard deviation of three technical replicates.

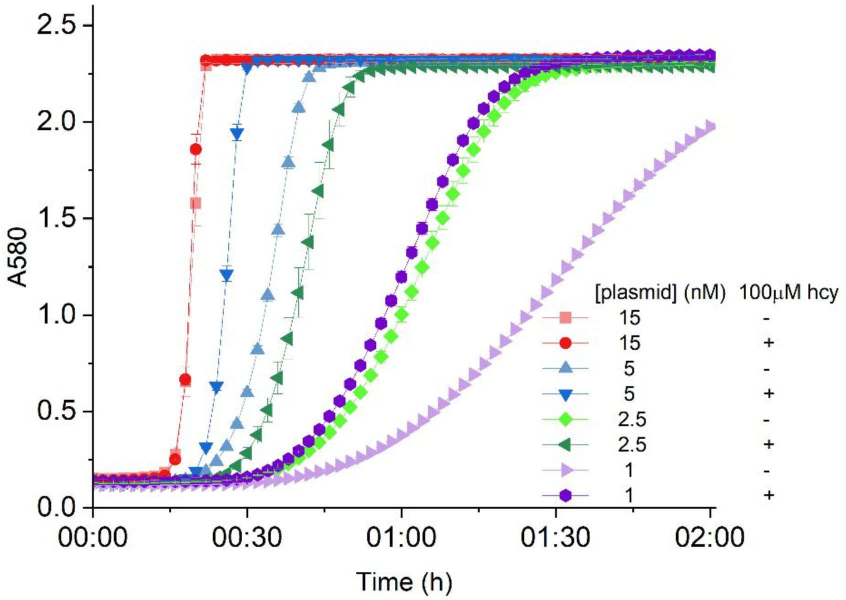

Figure S5. Colorimetric sensor response at different total plasmid concentrations in defined aqueous samples with saturating levels of homocysteine. As the total plasmid concentration is reduced from 15 nM, the differences in absorbance between reactions with and without 100 µM homocysteine are amplified, with maximal differences at 2.5 nM between 45 and 60 minutes. All reactions contained a 1:2 molar ratio of pMetR to reporter plasmid expressing LacZ. Data were collected during incubation at 37°C; error bars indicate the standard deviation of three technical replicates.

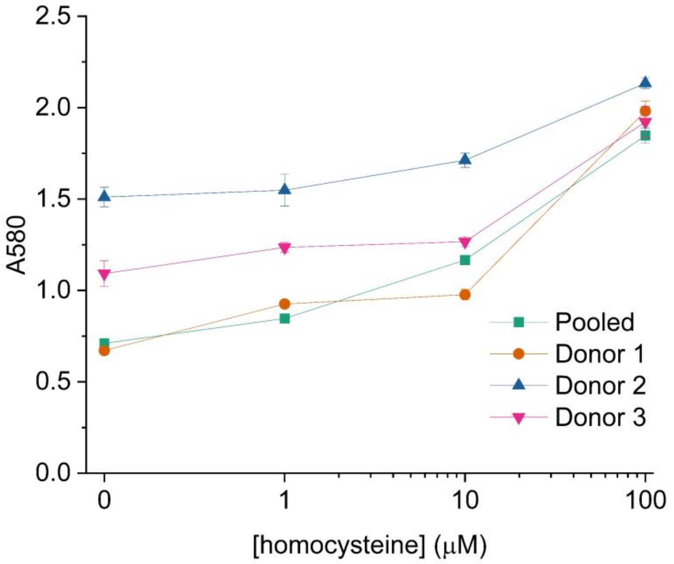

Figure S6. Colorimetric sensor response to homocysteine in pooled and single-donor serum samples at 40 minutes, when maximal differences in absorbance between successive measured concentrations were observed. Samples exhibit slightly different responses to homocysteine, indicating different homocysteine statuses and/or sample-specific effects. All reactions contain 0.83 nM pMetR and 1.67 nM of reporter plasmid expressing LacZ. Data were collected in 20% serum during incubation at 37°C; error bars indicate the standard deviation of three technical replicates. Homocysteine concentrations are the concentrations added to the serum sample before dilution and measurement.

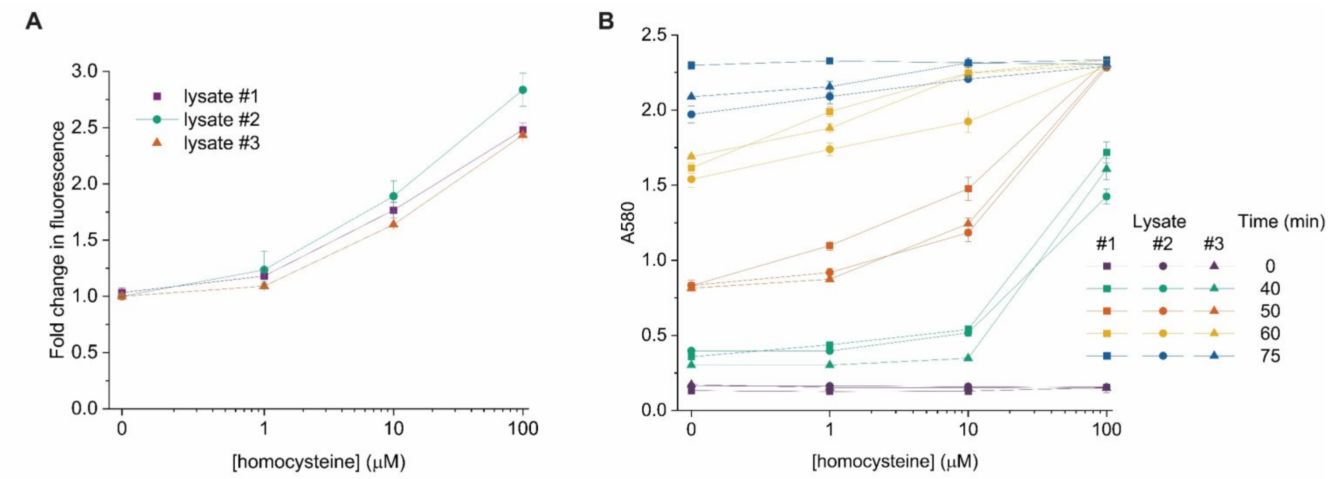

Figure S7. Variability in cell-free homocysteine sensing across different batches of lysate. **(A)** Sensor response with a sfGFP reporter in three lysate batches. Differences in sensor response are within what would be expected due to batch-to-batch variability in lysate strength. The experimental design corresponds with that of the data in Figure 1B, background fluorescence of cell-free reactions with no DNA template was subtracted from all samples, and data were collected after 4 h of incubation at 37 °C. **(B)** Sensor response with a LacZ-based colorimetric reporter in three lysate batches. Differences in color outputs between reactions using different batches of

lysate are likely not distinguishable with the naked eye, and sensing test results can be read at the same time (50 min). The experimental design corresponds with that of the data in Figure 2A, and data were collected during incubation at 37 °C. In both panels, lysate #1 is the batch previously used in Figures 1B and 2A, and error bars indicate the standard deviation of three technical replicates.

Table S1. Description of plasmid parts and DNA sequences in this paper

pMetEGFP	Plasmid encoding sfGFP expression under P_{MetE}
P_{MetE} Stability hairpin RBS sfGFP Terminator Kanamycin resistance cassette ColE1 origin<br><br>agatcaaaggatcttcttgagatcctttttttctgcgcgtaatctgctgcttgcaaacaaaaaaaccaccgctaccagcggtggtttgtttgcc<br>ggatcaagagctaccaactctttttccgaaggtaactggcttcagcagagcgcagataccaaatactgttcttctagtgtagccgtagttag<br>gccaccacttcaagaactctgtagcaccgcctacatacctcgctctgctaatcctgttaccagtggctgctgccagtggcgataagtcgtg<br>tcttaccgggttggactcaagacgatagttaccggataaggcgcagcggtcgggctgaacggggggttcgtgcacacagcccagcttg<br>gagcgaacgacctacaccgaactgagatacctacagcgtgagctatgagaaagcgccacgcttcccgaagggagaaaggcggaca<br>ggtatccggtaagcggcagggtcggaacaggagagcgcacgagggagcttccagggggaaacgcctggtatctttatagtcctgtcg<br>ggtttcgccacctctgacttgagcgtcgatttttgtgatgctcgtcaggggggcggagcctatggaaaaacgccagcaacgcgatcccg<br>cgaaatATAAATTAATAGCGTCTCGCTGGCGAGATCTTCCGGTGTAATTCGCGTTTTC<br>GCCGCCAGTGGATGGTCAGGTGCTAACACCAGACGCACTTCATAGTCGAACATCG<br>GCGAATAATGCAGGCCACTGCGCGGCAGAATATCGGACGTCATTACCAGATCCAG<br>CTCTCCCTGTTGCAAGGCGGGCTGCGGGTCAAATGTCACGCCCGATTTAAAATCCA<br>TCTCTACCTGCGGCCAGTTCTTATGGAAATTTTCTAACGCGGGTGTCAGCCACTGA<br>ATACAGCTATGGCACTCAATGGCAATGCGCAGACGCGTCTGCTGCGGTTCATTGC<br>AGGCTTGCAGGGCCTGGCTAATTTGCGGCAGTACCTGGTTTGCCAGTTGCAACAG<br>GATTTCTCCCTGCGGTGTAAAGCGTAGCGGCTGGCTCTTACGCACAAATAGCCGG<br>AAGCCAAGGCGTTGTTCCAGATCGCTAAACTGGTGAGACAGGGCGGATTGCGTCT<br>GATGCAACGTCGCCGCAGCGGCTGCGAGCGAGCCGCAGTTCCGCAACGCTTGTAG<br>CGTTTTCAGGTGTTTTACTTCGATCATGAAAGTCCTTCACTTCGGCATGAATAATTT<br>GCGCTTGAGGAATATACAGTAACCGCCAATTATGGATGTGTAAACATCTGGACGG<br>CTAAAATCCTTCGTCTTTTAAATTTATGGTGCGTTGGCTGCGTTTCTCCACCCCGGT<br>CACccacaacggtttccctctagaaataattttgtttaactttaagaaggagatatacatATGAGCAAAGGTGAAGAAC<br>TGTTTACCGGCGTTGTGCCGATTCTGGTGGAACTGGATGGCGATGTGAACGGTCAC<br>AAATTCAGCGTGCGTGGTGAAGGTGAAGGCGATGCCACGATTGGCAAACTGACGC<br>TGAAATTTATCTGCACCACCGGCAAACTGCCGGTGCCGTGGCCGACGCTGGTGAC<br>CACCCTGACCTATGGCGTTCAGTGTTTTAGTCGCTATCCGGATCACATGAAACGTC<br>ACGATTTCTTTAAATCTGCAATGCCGGAAGGCTATGTGCAGGAACGTACGATTAG<br>CTTTAAAGATGATGGCAAATATAAAACGCGCGCCGTTGTGAAATTTGAAGGCGAT<br>ACCCTGGTGAACCGCATTGAACTGAAAGGCACGGATTTTAAAGAAGATGGCAATA<br>TCCTGGGCCATAAACTGGAATACAACTTTAATAGCCATAATGTTTATATTACGGCG<br>GATAAACAGAAAAATGGCATCAAAGCGAATTTTACCGTTCGCCATAACGTTGAAG<br>ATGGCAGTGTGCAGCTGGCAGATCATTATCAGCAGAATACCCCGATTGGTGATGG<br>TCCGGTGCTGCTGCCGGATAATCATTATCTGAGCACGCAGACCGTTCTGTCTAAAG<br>ATCCGAACGAAAAAGGCACGCGGGACCACATGGTTCTGCACGAATATGTGAATGC<br>GGCAGGTATTACGTGGAGCCATCCGCAGTTCGAAAAATAAgtcgaccggctgctaacaaagccc<br>gaaaggaagctgagttggctgctgccaccgctgagcaataactagcataaccccttggggcctctaaacgggtcttgaggggttttttgc	

tgaaagccaattctgattagaaaaactcatcgagcatcaaatgaaactgcaatttattcatatcaggattatcaataccatatttttgaaaaag<br>ccgtttctgtaatgaaggagaaaactcaccgaggcagttccataggatggcaagatcctggtatcggtctgcgattccgactcgtccaac<br>atcaatacaacctattaatttcccctcgtcaaaaataaggttatcaagtgagaaatcaccatgagtgacgactgaatccggtgagaatggc<br>aaaagcttatgcatttctttccagacttgttcaacaggccagccattacgctcgtcatcaaaatcactcgcatcaaccaaaccgttattcattc<br>gtgattgcgcctgagcgagacgaaatacgcgatcgctgttaaaaggacaattacaaacaggaatcgaatgcaaccggcgcaggaaca<br>ctgccagcgcatcaacaatattttcacctgaatcaggatattcttctaatacctggaatgctgttttcccggggatcgcagtggtgagtaacc<br>atgcatcatcaggagtacggataaaatgcttgatggtcggaagaggcataaattccgtcagccagtttagtctgaccatctcatctgtaaca<br>tcattggcaacgctacctttgccatgtttcagaaacaactctggcgcatcgggcttcccatacaatcgatagattgtcgcacctgattgccc<br>gacattatcgcgagcccatttatacccatataaatcagcatccatgttggaatttaatcgcggcttcgagcaagacgtttcccgttgaatatg<br>gctcataacaccccttgtattactgtttatgtaagcagacagttttattgttcatgatgatatatttttatcttgtgcaatgtaacatcagagattttg<br>agacacaacgtg

pMetR	Plasmid encoding MetR expression under P_{T7}
P_{T7} Stability hairpin RBS met*R* Terminator Kanamycin resistance cassette ColE1 origin<br>agatcaaaggatcttcttgagatcctttttttctgcgcgtaatctgctgcttgcaaacaaaaaaaccaccgctaccagcggtggtttgtttgcc<br>ggatcaagagctaccaactctttttccgaaggtaactggcttcagcagagcgcagataccaaatactgttcttctagtgtagccgtagttag<br>gccaccacttcaagaactctgtagcaccgcctacatacctcgctctgctaatcctgttaccagtggctgctgccagtggcgataagtcgtg<br>tcttaccgggttggactcaagacgatagttaccggataaggcgcagcggtcgggctgaacggggggttcgtgcacacagcccagcttg<br>gagcgaacgacctacaccgaactgagatacctacagcgtgagctatgagaaagcgccacgcttcccgaagggagaaaggcggaca<br>ggtatccggtaagcggcagggtcggaacaggagagcgcacgagggagcttccagggggaaacgcctggtatctttatagtcctgtcg<br>ggtttcgccacctctgacttgagcgtcgatttttgtgatgctcgtcaggggggcggagcctatggaaaaacgccagcaacgcgatcccg<br>cgaaattaatacgactcactatagggagaccacaacggtttccctctagaaataattttgtttaactttaagaaggagatatacatatgatcg<br>aagtaaaacacctgaaaacgctacaagcgttgcggaactgcggctcgctcgcagccgctgcggcgacgttgcatcagacgcaatccg<br>ccctgtctcaccagtttagcgatctggaacaacgccttggcttccggctatttgtgcgtaagagccagccgctacgctttacaccgcaggg<br>agaaatcctgttgcaactggcaaaccaggtactgccgcaaattagccaggccctgcaagcctgcaatgaaccgcagcagacgcgtctg<br>cgcattgccattgagtgccatagctgtattcagtggctgacacccgcgttagaaaatttccataagaactggccgcaggtagagatggatt<br>ttaaatcgggcgtgacatttgacccgcagcccgccttgcaacagggagagctggatctggtaatgacgtccgatattctgccgcgcagt<br>ggcctgcattattcgccgatgttcgactatgaagtgcgtctggtgttagcacctgaccatccactggcggcgaaaacgcgaattacaccg<br>gaagatctcgccagcgagacgctattaatttatccggtgcagcgtagtcgactggatgtctggcggcattttcttcagccggcaggcgtc<br>agcccgtcactgaaaagcgtcgataacaccttattgttgattcagatggttgccgcgcggatgggtattgccgcgctaccgcattgggta<br>gtagagagttttgagcgccagggtctggtggtgaccaaaaccctgggcgaaggcttgtggagccgactgtacgccgccgtgcgcgat<br>ggcgagcagcgtcagccagttacggaagcgtttattcgctcagcgcgcaatcacgcctgcgatcatctgccgtttgtgaagagcgcgg<br>agcgacccacttacgatgcacccacagtgaggccaggatcaccagcgcgcctgtaagtcgaccggctgctaacaaagcccgaaagg<br>aagctgagttggctgctgccaccgctgagcaataactagcataaccccttggggcctctaaacgggtcttgaggggttttttgctgaaagc<br>caattctgattagaaaaactcatcgagcatcaaatgaaactgcaatttattcatatcaggattatcaataccatatttttgaaaaagccgtttct<br>gtaatgaaggagaaaactcaccgaggcagttccataggatggcaagatcctggtatcggtctgcgattccgactcgtccaacatcaatac<br>aacctattaatttcccctcgtcaaaaataaggttatcaagtgagaaatcaccatgagtgacgactgaatccggtgagaatggcaaaagctt<br>atgcatttctttccagacttgttcaacaggccagccattacgctcgtcatcaaaatcactcgcatcaaccaaaccgttattcattcgtgattgc<br>gcctgagcgagacgaaatacgcgatcgctgttaaaaggacaattacaaacaggaatcgaatgcaaccggcgcaggaacactgccag<br>cgcatcaacaatattttcacctgaatcaggatattcttctaatacctggaatgctgttttcccggggatcgcagtggtgagtaaccatgcatc<br>atcaggagtacggataaaatgcttgatggtcggaagaggcataaattccgtcagccagtttagtctgaccatctcatctgtaacatcattgg<br>caacgctacctttgccatgtttcagaaacaactctggcgcatcgggcttcccatacaatcgatagattgtcgcacctgattgcccgacatta<br>tcgcgagcccatttatacccatataaatcagcatccatgttggaatttaatcgcggcttcgagcaagacgtttcccgttgaatatggctcata<br>acaccccttgtattactgtttatgtaagcagacagttttattgttcatgatgatatatttttatcttgtgcaatgtaacatcagagattttgagacac<br>aacgtg	

Table S2. Primers used for amplification from *Escherichia coli* DH10B genomic DNA template. Uppercase sequences indicate primer annealing regions. Lowercase sequences indicate overhang sequences needed for Gibson assembly.

**Amplicon**	**Primers**
P_{MetE}	For cloning into pMetRGFP:<br>Forward: ATAAATTAATAGCGTCTCGCTGGC<br><br>Reverse: GTGACCGGGGTGGAGAA
metR	For cloning into pMetR:<br>Forward:<br>aactttaagaaggagatatacatATGATCGAAGTAAAACACCTGAAAACGCT<br><br>Reverse: ctttgttagcagccggtcgacTTACAGGCGCGCTGGTG<br>tgaactatcatgaaggtaaatggcgTGGCGCCGATGGGCGCCAttaGCAGATTGTTTT<br>TTCTTCAATGAAC
P_{GlyA}	Forward: GCCGCATGGAACCAGTTC<br><br>Reverse: CCGCATGGAACCAGTTC
